# Impact of Classical Risk Factors on Subclinical Carotid Atherosclerosis Progression: Insights from a Non-Diabetic Cohort

**DOI:** 10.31083/j.rcm2503103

**Published:** 2024-03-12

**Authors:** Eva Szabóová, Alexandra Lisovszki, Peter Kolarčik, Eliška Fatĺová, Tomáš Molnár, Martin Bujdoš, Peter Szabó

**Affiliations:** ^1^Department of Angiology, Faculty of Medicine, Pavol Jozef Šafárik University in Košice and East Slovak Institute of Cardiovascular Diseases, 040 11 Košice, Slovakia; ^2^4th Department of Internal Medicine, Faculty of Medicine, Pavol Jozef Šafárik University in Košice and Louis Pasteur University Hospital, 041 90 Košice, Slovakia; ^3^Department of Health Psychology and Research Methodology, Faculty of Medicine, Pavol Jozef Šafárik University in Košice, 040 11 Košice, Slovakia; ^4^Department of Cardiac Surgery, Faculty of Medicine, Pavol Jozef Šafárik University in Košice and East Slovak Institute of Cardiovascular Diseases, 040 11 Košice, Slovakia; ^5^Department of Angiology, Cardiocentrum AGEL Košice-Šaca, 040 15 Košice, Slovakia; ^6^Faculty of Aeronautics, Technical University of Košice, 041 21 Košice, Slovakia

**Keywords:** subclinical atherosclerosis, atherosclerotic plaque, intima-media thickness, cardiovascular risk factors, risk score

## Abstract

**Background:**

Several markers have been proposed for the detection and progression of subclinical atherosclerosis. We aimed to analyse the impact of classical risk factors on the presence and short-term progression of subclinical carotid atherosclerosis in a non-diabetic, primary prevention cohort.

**Methods:**

This analysis included participants with completed visits at baseline and at 5-year follow-up (N = 141; 56.7% females, 43.3% males; aged 49.6 ± 4.7 years). Clinical and laboratory parameters, risk profiles, carotid artery intima-media thickness (CIMT) and plaque presence were analysed.

**Results:**

There was a significant progression in mean CIMT (0.54 ± 0.09 mm–0.62 ± 0.10 mm; *p* < 0.001), prevalence of carotid plaque (4.8%–17.9%; *p* < 0.001) and age- and sex-adjusted abnormal CIMT (52.9%–78.8%; *p* < 0.001) at the end of follow-up, compared to baseline. In multivariate regression analysis, among the classical risk factors, their number, metabolic syndrome and SCORE (Systematic Coronary Risk Estimation) risk only the number of risk factors showed an independent and significant impact on the occurrence of a carotid plaque (Exp(B) = 1.71; *p* = 0.017) and 5-year CIMT progression.

**Conclusions:**

During a short follow-up, the significant progression of subclinical atherosclerosis was confirmed. The number of risk factors predicted the occurrence of carotid plaques and CIMT progression. The high prevalence and short-term progression of subclinical carotid atherosclerosis underly the rationale for its screening in personalized cardiovascular risk stratification in asymptomatic middle-aged subjects over 50 years old, at low-to moderate cardiovascular risk, particularly with several risk factors.

## 1. Introduction

Atherosclerotic (ATS) cardiovascular (CV) diseases (CVD) are the main cause of morbidity and mortality worldwide [1]. The presence of subclinical ATS is the major causal risk factor (RF) for CVD in asymptomatic individuals [1]. Various markers of subclinical ATS have been identified as predictors of CV events [1]. In contrast, the management of patients without established CVD is based solely on the identification of the risk of CVD, mostly through validated multivariable risk prediction tools. However, the calculated risk may underestimate the real CVD risk [1, 2]. A large proportion of the asymptomatic population assessed by risk scoring is at low-to moderate CVD risk, with missed opportunities for early detection and appropriate management of CVD [3, 4]. The use of biochemical, functional, and morphological markers of subclinical ATS was proposed to refine the risk classification in subjects with low- to moderate CV risk profiles [2, 5]. Morphological changes of the arterial wall, detected by coronary artery calcification (CAC), carotid artery intima-media thickness (CIMT) and carotid plaque detection were shown to be the most valuable markers of subclinical ATS and predictors of CV events, however, not with equal risk reclassification [6]. CAC is a surrogate measure of total ATS plaque burden and a strong independent predictor of CV morbidity and mortality but has significant limitations for primary prevention [6, 7]. In contrast, detection of CIMT and carotid plaque can be easily measured at a reduced cost, without radiation, but with lower net reclassification value than CAC-scoring [6]. Systematic reviews [7, 8] have documented that CAC scoring, CIMT and the presence of carotid plaque improved risk prediction in addition to traditional risk scores in the low to-intermediate risk population, with CAC being the best measure, followed by CIMT and carotid plaque quantification [9, 10, 11]. However, carotid plaque quantification offers better accuracy and reproducibility in assessing subclinical ATS compared to CIMT [8]. Several sophisticated, promising imaging markers for identifying subclinical ATS and improving risk stratification in asymptomatic subjects are available, however, the lack of methodological standardization, measurement difficulties and publication bias argue against screening [3, 12]. Therefore, the current European guidelines suggest not using genetic risk scores, circulating or urinary biomarkers, vascular tests or imaging methods (other than CAC scoring or carotid ultrasound (USG)) for risk estimation [13]. CAC scoring, or plaque detection by carotid USG when CAC scoring is not feasible, may be considered to improve risk classification for treatment decisions with a IIb B level of evidence [13].

ATS progression predicts CV events [14]. However, existing data regarding the association between progression of carotid intima-media thickness (IMT) and the risk of CV events remains inconclusive [14, 15, 16]. No reliable data from the literature is available on the rate of progression of pathological age and sex adjusted CIMT. Conflicting data also exists on the short and long-term influence of CVD risk profiles on the progression of carotid ATS [17].

We aimed to study the prevalence and short-term progression of subclinical carotid ATS in middle-aged, non-diabetic, asymptomatic individuals with low-to moderate estimated CV risk as well as to evaluate the associations between CV RFs and morphological markers. Our secondary aim was to show the efficacy of carotid plaque screening for personalized CV risk stratification. To the best of our knowledge, only a few studies have combined carotid IMT parameters and the presence of plaque to study the progression of subclinical carotid ATS in a middle-aged, non-diabetic, primary prevention cohort.

## 2. Patients and Methods

This was an observational, prospective, real-life study in a population of 400–450 asymptomatic subjects, based mainly on loco-regional specificity. The study subjects were 141 participants of Caucasian origin without established CVD, 80 (56.7%) females and 61 (43.3%) males, aged 49.6 ± 4.7 years, who underwent 2 visits, at baseline and at the end of 5-year follow-up (4.67 ± 0.95 years) between February 2010 and October 2017. We invited subjects to participate in the study for the purpose of screening subclinical ATS through various social media platforms. We included patients: aged 35–55 years, non-diabetics, from the Košice region, with signed an informed consent. We excluded patients with: established CVD, European SCORE (Systematic Coronary Risk Estimation) risk ≥5% (to select underdiagnosed and undertreated subjects) [1, 13], chronic kidney disorders (CKD) including estimated glomerular filtration rate (eGFR) <60 mL/min/$m^{2}$ as well as pathological urinary findings, neoplastic, hepatic, and chronic respiratory disorders, severe obesity, (body mass index (BMI) >35 kg/$m^{2}$), heavy alcohol use (for women/men consuming ≥4/≥5 drinks on any day or ≥8/≥15 drinks per week), non-compliance with treatment instructions, pregnancy, and acute inflammatory disorders. Out of the target population, 256 persons met the inclusion criteria, and 69 patients were excluded, mainly due to high SCORE risk, history of diabetes/baseline results confirming diabetes mellitus (DM) (fasting glucose, glycated haemoglobin (HbA1c)) [1, 18], severe obesity and renal abnormalities. Finally, 187 individuals were enrolled into the study, of which 141 (75,4%) completed the entire follow-up period. During follow-up, we observed one sudden cardiac death (0.53%), one suicidal death, and one nonfatal CV event (unstable angina pectoris). The study protocol was approved by the Ethical Committee of the LP University Hospital in Košice.

## 3. Data Collections and Statistics

### 3.1 Data Collections

Participants were examined in the Outpatient Department of the 4th Clinic of Internal Medicine at LP University Hospital in Kosice. The examinations were carried out in the morning, under standard conditions. We performed blood sampling, urine tests, electrocardiograms, subclinical ATS markers, and conducted medical interviews to detect major RFs for ATS and pharmacotherapy. On physical examination, anthropometric parameters and office blood pressure were measured. A 10-year total CV mortality (SCORE) and Framingham risk score were calculated for each subject. Blood and urine samples were analysed by standard laboratory methods. Metabolic parameters included: fasting glucose, glycated haemoglobin (HbA1c), uric acid, serum total cholesterol (T-C), high-density lipoprotein cholesterol (HDL-C), low-density lipoprotein cholesterol (LDL-C), triglycerides (TAG) and serum creatinine. The estimated glomerular filtration rate was calculated according to the “Modification of Diet in Renal Disease” formula [19]. The following values were considered abnormal for the primary prevention cohort, based on accredited laboratory reference values: T-C >5.0 mmol/L, LDL-C >3.0 mmol/L, HDL-C <1.0/<1.2 mmol/L (males/females), TAG >1.7 mmol/L [20], creatinine >90 µmol/L, eGFR <90 mL/min/$m^{2}$, uric acid >357/428 µmol/L (males/females). Non-modifiable RFs for ATS as well as arterial hypertension (AH), dyslipoproteinemia (DLP), obesity/central obesity, diabetes mellitus (DM), impaired fasting glucose, metabolic syndrome (MetS) were defined according to current recommendations [1, 18]. Smoking status was characterized as current smoking ≥1 cigarette/day. To compute an individual’s 10-year fatal risk, the SCORE charts for high-risk countries, applicable to Slovakia (low risk <1%/moderate risk ≥1% and <5%/high risk ≥5% and <10%/very high risk ≥10%) were used. The total CV event risk was computed as follows: fatal risk × 3(4) for males (females). The SCORE system estimates the 10-year risk of a first fatal atherosclerotic CVD in populations of countries at low/high/very high CV risk, using sex, smoking status, age, systolic blood pressure and T-C as variables [1]. For comparison, the 10-year risk for hard coronary events (derived from the Framingham Heart Study—FHS) was also calculated, using age, sex, T-C, HDL-C, smoking status, DM, systolic blood pressure, and hypertensive treatment. FHS scores were classified as low (<10%), moderate (10–20%), or high (<20%) risk [21]. Based on the latest CVD prevention guidelines [13], we refined the calculated SCORE 10-year fatal CV risk by using carotid plaque burden to specify the personalized CV risk at baseline and after follow-up. The targeted dietary and pharmacological management of AH and DLP was satisfactory at the enrolment visit, no polypharmacotherapy was observed, and subjects were mainly treated with one prescribed drug. Based on personalized CV risk assessment, preventive measures (predominantly lifestyle modifications, or individually, if it was indicated according to the results on regular clinical and laboratory check-ups, pharmacological treatment) were recommended for each subject, to which they agreed. Adherence to treatment instructions was regularly checked by family doctors and by the study investigators at the end of follow-up.

### 3.2 Morphological Markers of Subclinical Carotid Atherosclerosis. Carotid IMT and Plaque Assessment

Carotid arteries were examined using ultrasonography (USG) by the same experienced physician for all patients and all years and were blinded to the subjects’ health status and RFs. USG methodology as well as CIMT and carotid plaque definitions followed the Mannheim consensus and European recommended protocols [22, 23]. Bilateral carotid arteries were scanned using high-resolution B-mode USG (Philips HD 15) with the 7.5-MHz probe in real-time, at 5× magnification. IMT was measured automatically, on distinct plaque-free common carotid artery (CCA) posterior wall, 10 mm proximal to the flow divider, during end-diastole, at its presumed maximum thickness. We used the mean of 4 measured values for each side. The definition of a plaque was a focal wall protrusion into the arterial lumen of at least 1.5 mm or >50% of the surrounding IMT value. Plaques were recorded in transverse and longitudinal planes in 4 segments (CCA, bulb, internal carotid artery (ICA), external carotid artery (ECA)). No patient had significant carotid plaque with pathological peak systolic velocity (PSV) acceleration. Generally, carotid plaques were stable, with smooth surface and were isoechogenic at baseline and during follow-up. CCA parameters evaluated in our study included: mean right, left IMT (CIMTdx, sin), maximum IMT (CIMTmax), right or left IMT >0.9 mm (CIMTbilat >0.9) [24], abnormal age and sex adjusted mean right or left IMT (asCIMTbilat), i.e., in males/females on the left side, aged in years (y): 31–40 y: 0.57/0.51 mm, 41–50 y: 0.61/0.57 mm, >50 y: 0.70/0.64 mm; on the right side: 31–40 y: 0.5/0.49 mm, 41–50 y: 0.57/0.53 mm, >50 y: 0.62/0.59 mm [25, 26], CCA-IMT progression (mm/year), Δ CIMT (difference in mean CIMT during follow-up) and presence of carotid plaque. Due to the identical progression rate and values (mean ± SD) of CIMT on both sides at 2 visits, for statistical analysis we used CIMTsin. The rate of IMT change per year was calculated by using the change of mean CCA IMT (between end and baseline of the study) divided by the time interval between the two ultrasound scans (approximately 5 years) [16]. Our intra-observer variability was acceptable (mean absolute difference = 0.085 ± 0.069; correlation coefficient = 0.88; coefficient of variation = 7.2%).

### 3.3 Statistical Analysis

Patient’s data are summarized at baseline and at the end of follow-up. Continuous variables were presented as mean ± standard deviation (SD), categorical variables as the number of cases with percent frequency. Continuous clinical variables, including markers of subclinical carotid ATS between patients at baseline and at follow-up visit were compared using a paired samples *t*-test. A McNemar’s test was used to compare the frequencies of categorical variables in time between paired samples. The crude effect of traditional RF on dichotomized/continuous markers of carotid subclinical ATS was tested by binary logistic/linear regression analysis. Normal distribution of selected parameters was confirmed in CIMTdx and CIMTsin, however, our distribution tends to be bimodal or multimodal and still followed a normal curve on the graph. Crude effect of categorical (positive family history for CVD, CV risk age: in men/women ≥45/≥55 years [27], sex, AH, DLP, smoking, central obesity, MetS) and continuous (duration of AH, T-C, HDL-C, LDL-C, TAG, number of RFs, SCORE) were assessed using univariate regression analysis. Correlations were tested with follow-up parameters, with suspected higher prevalence of subclinical ATS markers. In multivariate analysis we tested a mutually adjusted influence of all predictors from the univariate model on the analysed morphological parameters. Significant predictors of subclinical carotid ATS were established from the multivariate model using the backward (Wald) method. Parameters used in the univariate analysis were entered into the multivariate model. Pharmacotherapy as a covariate was not tested in the study due to the wide spectrum of analysed predictors and relatively small number of subjects on medication. During the follow-up a progression rate of CIMT was also calculated. The changes in computed (using SCORE charts) and personalized CV risk stratification were also compared at baseline and after follow-up. A value of *p* < 0.05 was considered significant. Data analyses were performed in IBM SPSS 23.0 Statistics (IBM Corp., Armonk, NY, USA).

## 4. Results

### 4.1 The Prevalence of Risk Factors and Subclinical Carotid Atherosclerosis at Baseline and at Follow-up 

Out of the 187 initial, healthy, non-diabetic, 35–55-year-old (mean age 45.6 ± 5 years at baseline) subjects, 141 persons were examined after a follow-up of 4.67 ± 0.95 years. The characteristics of the study population at baseline and after follow-up are shown in Table 1. Worsening of the persons’ risk profile within 5 years is documented in Table 2. After follow-up we documented a significantly higher prevalence of DLP, central obesity, AH, as well as the number of RFs (3.78 ± 6.06; *p* < 0.05). The risk SCORE and FHS score were increased from baseline to the end of follow-up (0.57 ± 0.93–1.16 ± 1.56 and 3.66 ± 4.73–6.18 ± 6,84, respectively), but still remained at a low-moderate risk level. Changes in analysed markers of subclinical carotid ATS during follow-up are listed in Table 3. The mean values of CIMT left and right (0.62 ± 0.10 mm; *p* < 0.001, both) remained under the cut off level of 0.9 mm at follow-up but were significantly increased (by 0.08 ± 0.11–0.12 mm; *p* < 0.001). A similar significant increase in maximum CIMT values (0.08 ± 0.12 mm; *p* < 0.001) was also detected. The mean right and left CCA-IMT change/year was the same: 0.017 mm. The occurrence of CIMT >0.9 mm was rare (2.1%) and not significantly altered. However, the prevalence of asCIMTbilat was significantly increased from 52.9% to 78.8% (*p* < 0.001). A similar increase in the rate of carotid plaque burden in the CCA, bulb, ICA and ECA was also observed (from 4.8% to 17.9%; *p* < 0.001).

**Table 1.** **Comparison of mean values, standard deviations (SDs) and changes (Δ) of continuous demographic, clinical and laboratory parameters at baseline and after follow-up assessed with paired *t*-test**.

Parameters	Baseline	Follow-up	Δ	*p*
	N = 141 Mean (SD)	N = 141 Mean (SD)	Mean (SD)	
Age (yr)	45.64 (5.02)	49.64 (4.67)	4.35 (1.6)	<0.001
Waist circumference (cm)	87.63 (13.07)	92.33 (12.87)	4 (5.39)	<0.001
BMI (kg/$m^{2}$)	25.28 (3.89)	25.67 (4.55)	0.38 (1.48)	0.003
Total cholesterol (mmol/L)	5.47 (0.93)	6.00 (1.09)	0.48 (0.88)	<0.001
LDL-C (mmol/L)	3.24 (0.79)	3.91 (0.83)	0.63 (0.75)	<0.001
HDL-C (mmol/L)	1.5 (0.35)	1.47 (0.36)	–0.01 (0.21)	NS
Triglycerides (mmol/L)	1.26 (0.74)	1.47 (0.856)	0.15 (0.56)	0.002
Plasma glucose (mmol/L)	5.01 (0.47)	5.13 (0.49)	0.11 (0.4)	0.001
HbA1c (IFCC) (mmol/mol)	34.4 (3.6)	32.4 (3.5)	–1.9 (3.4)	<0.001
Uric acid (µmol/L)	297.27 (80.09)	312.16 (81.9)	13.97 (45.31)	0.001
Creatinine (µmol/L)	86.45 (10.64)	71.36 (11.91)	–16.36 (5.63)	<0.001
eGFR (mL/min/1.73 $m^{2}$)	70.2 (7.8)	96.6 (11.4)	26.4 (9.0)	<0.001

Remarks: BMI, body mass index; LDL-C, low-density lipoprotein cholesterol; HDL-C, high-density lipoprotein cholesterol; HbA1c, glycated haemoglobin; IFCC, International Federation of Clinical Chemistry and Laboratory Medicine; eGFR, estimated glomerular filtration rate; NS, statistically nonsignificant difference; N, number; SD, standard deviation; yr, years; Δ, change; *p*, statistical significance.

**Table 2.** **Comparison of prevalence and mean values of variables related to cardiovascular risk profile at baseline and after follow-up assessed with McNemar’s or paired *t*-test**.

Parameter	Baseline	Follow-up	*p*
	N = 187/141 Mean (SD)	N = 141 Mean (SD)	
Risk age (N/%)	41/21.9	65/46.1	NS**
Sex (male) (N/%)	75/40.1	61/43.3	NS**
Positive family history (N/%)	33/17.8	31/22.1	NS**
DLP (N/%)	132/71	126/89.4	<0.001**
AH (N/%)	48/25.8	54/38.6	<0.001**
Duration of AH (years)	0.78 (2.12)	2.1 (4.57)	<0.001*
Smoking (N/%)	38/20.3	28/19.9	NS**
MetS (N/%)	31/16.8	40/28.4	NS**
Central obesity (N/%)	105/57.4	103/74.6	<0.001**
SCORE fatal	0.57 (0.93)	1.16 (1.56)	<0.001*
SCORE non-fatal	1.81 (2.70)	3.71 (4.72)	<0.001*
Number of RF	2.61 (1.63)	3.78 (6.06)	<0.027*
Treatment of DLP (N/%)	12 (6.4)	12 (8.5)	NS**

Remarks: SCORE, Systematic Coronary Risk Estimation; DLP, dyslipoproteinemia; AH, arterial hypertension; MetS, metabolic syndrome; RF, risk factor; SD, standard deviation; NS, statistically nonsignificant difference; N, number; *p*, statistical significance; *, paired *t*-test (N = 141 at baseline and follow-up); **, McNemar’s test (N = 187 at baseline and N = 141 at follow-up).

**Table 3.** **Morphological markers of subclinical carotid atherosclerosis at baseline and after follow-up assessed with McNemar’s or paired *t*-test**.

Markers	Baseline	Follow-up	Δ	*p*
	N = 141 Mean (SD)	N = 141 Mean (SD)	Mean (SD)	
CIMT dx (mm)	0.54 (0.09)	0.62 (0.10)	0.08 (0.12)	<0.001
CIMT sin (mm)	0.54 (0.09)	0.62 (0.10)	0.08 (0.11)	<0.001
CIMT max (mm)	0.67 (0.11)	0.74 (0.11)	0.08 (0.12)	<0.001
CIMT bilat >0.9 (N/%)	2/1.1	3/2.1	1/1.0	NS*
asCIMT bilat (N/%)	99/52.9	111/78.8	12/25.9	<0.001*
Carotid plaque (N/%)	9/4.8	25/17.9	16/13.1	<0.001*

Remarks: CIMT dx/sin/max, common carotid artery intima-media thickness: mean value of right/left carotid artery/maximum value; CIMT bilat >0.9 mm, common carotid artery intima-media thickness >0.9 mm bilaterally; asCIMT bilat, pathological common carotid artery intima-media thickness by age and sex on the right or left; SD, standard deviation; NS, statistically nonsignificant difference; N, number; *p*, statistical significance; *, McNemar’s test; Δ, change, difference. In McNemar’s test the sample size was N = 187.

### 4.2 The Influence of Cardiovascular Risk Factors on the Presence of Subclinical Carotid Atherosclerosis 

Based mainly on the surprisingly high prevalence of asCIMTbilat and carotid plaque burden in middle-aged, healthy population, we analysed the possible associations between classical RFs for ATS and carotid USG parameters, to determine their suitability as screening tests for subclinical ATS and eventually for personalized CV risk prediction. Statistically inconsistent associations were found (no relationship; borderline/weak significance) between classical RFs and CIMT parameters either continuous or dichotomous (including the mean CIMT difference between baseline and follow-up) in the univariate analysis (data are not shown). In contrast, a strong relationship was confirmed between all classical RFs (except for sex and positive family history) and the occurrence of carotid plaque (Table 4). Insignificant predictors are not shown. In the multivariate analysis we assessed the predictive power of the influence of classical RFs on markers showing progression of subclinical ATS. Total cholesterol was the only factor with a significant effect on the mean CIMT (*p* = 0.013; B = 0.024). A significant, but inconclusive influence of 2 lipid parameters was also detected on the pathological value of age- and sex-adjusted CIMT bilaterally (T-C: *p* = 0.019; Exp(B) = 58; LDL-C: *p* = 0.044; Exp(B) = 0.017). The multivariate regression model from the CIMT max showed statistical significance in non-lipid variables: CV risk score (*p <* 0.001; B = 0.028) and male sex (*p* = 0.002; B = –0.087). Nonetheless, the higher number of RFs (as the only independent variable) increased the probability of plaque occurrence on carotid arteries by 1.7 (Table 5). The number of RFs was a weak determinant of individual 5-year CIMT progression (Table 5). In comparison with risk charts, by using the personalized approach (carotid plaque presence in addition to computed risk), the high CV risk was 4–5× more prevalent at baseline and at follow-up. At baseline, every subject was at low-to moderate (<5%) calculated 10-year fatal CV (SCORE) and hard coronary event (FHS) CV risk. By detecting carotid plaque, in 4.8% of subjects the CV risk was reclassified into high. After 5-year follow-up, high CV risk according to the SCORE chart was present in 4.3% of subjects, but using personalized stratification, the prevalence of high CV risk was 17.9%. Due to the low number of individuals on hypolipidemic treatment at the time of enrollment, we did not monitor the effect of hypolipidemic treatment on the progression of carotid ATS.

**Table 4.** **Univariate (crude) effect of risk factors for atherosclerosis as predictors on the presence of carotid plaque (dependent variable). Logistic regression coefficient Exp(B) and 95% Confidence Interval**.

Dependent parameters	Independent parameters	Exp(B)	Confidence Interval 95%		*p*
			Lower bound	Upper bound	
Carotid plaque	Risk age	3.86	1.49	9.97	0.005
	AH	2.88	1.19	7.02	0.019
	Smoking	3.59	1.40	9.24	0.008
	Central obesity	9.0	1.16	69.71	0.035
	MetS	3.53	1.44	8.64	0.006
	SCORE	1.44	1.12	1.85	0.004
	T-C	1.88	1.24	2.83	0.003
	TAG	1.57	0.99	2.49	0.057
	LDL-C	2.31	1.34	4.01	0.003

Remarks: AH, arterial hypertension; MetS, metabolic syndrome; T-C, total cholesterol; LDL-C, low density lipoprotein cholesterol; TAG, triglycerides; HDL-C, high-density lipoprotein cholesterol; SCORE, Systematic Coronary Risk Estimation; *p*, statistical significance. Variables at follow-up were entered into the logistic regression analysis. Dichotomic variables had two distinct alternatives (yes/no). Carotid plaque entered the analysis at N = 25 (every entered subject had only one plaque). Significant predictors are shown (sex, positive family history, duration of arterial hypertension, HDL-C and number of risk factors did not have significant association with carotid plaque).

**Table 5.** **Multivariate linear regression analysis from statistically significant predictors of left and maximum common carotid IMT, ΔCIMT as well as binary logistic regression analysis from statistically significant predictors of pathological values of age and sex adjusted common carotid IMT right or left and carotid plaque. Linear/logistic regression coefficient (B/Exp(B)) and 95% confidence interval**.

Dependent parameters	Independent parameters	B/Exp(B)	Confidence Interval 95%		*p*
			Lower bound	Upper bound	
CIMT sin	T-C	0.024	0.005	0.042	0.013
CIMT max	Sex (male)	–0.087	–0.14	–0.034	0.002
	SCORE	0.028	0.014	0.042	<0.001
asCIMT bilat	T-C	58*	1.94	1752.41	0.019
	LDL-C	0.017*	0.0003	0.902	0.044
ΔCIMT	Number of RFs	0.033	0.012	0.055	0.003
	Positive FH	–0.075	–0.134	–0.017	0.013
Carotid plaque	Number of RFs	1.71*	1.099	2.68	0.017

Remarks: SCORE, Systematic Coronary Risk Estimation; CIMT, carotid artery intima-media thickness; IMT, intima-media thickness; CIMT sin/max, common carotid artery intima-media thickness: mean value of left carotid artery/maximum value; asCIMT bilat, pathological common carotid artery intima-media thickness by age and sex on the right or left; ΔCIMT, difference in mean left common carotid artery intima-media thickness during follow-up; RFs, risk factors; FH, family history; T-C, total cholesterol; LDL-C, low density lipoprotein cholesterol; B, linear regression coefficient; *, logistic regression coefficient Exp(B); *p*, statistical significance. Due to listwise elimination of cases with missing cases there was a reduction in sample size in multivariate analysis (The actual sample size of CIMT sin/max/asCIMT bilat N = 86, for ΔCIMTsin N = 84, for carotid plaque N = 80. Positive cases: 36× CIMTsin and CIMT max, 64× asCIMT bilat, 13× carotid plaque).

## 5. Discussion

In clinically healthy, middle-aged, nondiabetic, predominantly non-hypertensive individuals, without known CVD, with low-to moderate estimated risk SCORE, during 5-year follow-up, the increase in mean and maximum values of CIMT was significant. The occurrence of age- and sex-adjusted abnormal mean CIMT was surprisingly high at the end of follow-up (78.8%) and compared to the beginning of the study, the prevalence was higher by 25.9%. Similarly, a relatively high prevalence (17.9%) of carotid plaque burden with a 13.1% increase in comparison with baseline was documented at the end of follow-up. Over 5 years, 95.7% of the study group remained at low- to moderate estimated CV risk (SCORE), in 4.3% of subjects a high risk was computed. Following personalized stratification, using carotid plaque for subclinical ATS detection, 13.6% of subjects were reclassified into high CV risk. These findings underline the role of timing (49.6 ± 4.7 years of age at the end of study) for population screening in terms of cost/benefit relations (4.8% vs. 13.6% reclassified CV risk at age 45 vs. 50 years, respectively). While RFs showed weak correlations with CIMT parameters in univariate and multivariate analysis, correlations were strong for the presence of carotid plaque. The number of RFs (as the only independent variable) increased the probability of plaque occurrence on carotid arteries by 1.7 and was a weak determinant of individual 5-year CIMT progression.

### 5.1 Risk Profile

The risk profile of our study group is comparable with the literature [17, 28], but obesity and DLP are increased in our study due to the fact, that we followed central obesity and had tighter cut-offs for DLP. In the large on-going PESA study with enrollment of participants without CVD, with no exclusion of diabetics, the study group had a better risk profile in term of DLP (40.9%) and obesity (13.3%), but the proportion of lipid-lowering therapy was similar (6.6%) [29].

### 5.2 CIMT Progression

Based on a systematic review, in low-to-intermediate risk individuals (mean age of 60 ± 7.6 years) the mean CIMT varied between 0.62–1.07 mm, and CIMTmax between 0.78–1.8 mm [7]. In the large ongoing Progression of Early Subclinical Atherosclerosis (PESA) study, similar to our results with a similar mean age, the mean CIMT value was 0.59 mm [4, 29]. The progression rate of the mean (SD) CCA-IMT in our study was in line with published data, with some limitations due to varying progression rates for CIMT reported in different population-based studies, ranged between 0.0038–0.060 mm/year [30, 31]. Comparable progression rates were reported in other studies [16, 32]. A mildly higher rate of CCA-IMT (0.025 mm/year) was observed in the large ARIC study [33]. The CAPS study reported a 0.001 mm/year progression rate of the CCA-IMT [17]. Increased CIMT represents subclinical vascular disease, may be related to intimal or medial hypertrophy or both, and may be an adaptive response to changes and is not clearly synonymous with subclinical ATS but is related to it due to similar alterations in the progression of both processes [34]. CIMT in subclinical vascular disease is a marker of CVD risk [35].

For CVD risk assessment, instead of normative values (i.e., pathological IMT >0.9 mm, reflecting primarily ATS at the carotid bifurcation and hypertension mediated hypertrophy at the level of CCA), carotid USG imaging and measurements should follow protocols with CIMT values in percentiles by age, sex, race/ethnicity and mostly also by side [25, 26, 36]. In comparison with previous data [28, 37], the occurrence of CIMT >0.9 mm was rare in our study and not significantly changed after 5-year follow up. CIMT >0.9 mm was detected in 1% of participants in the PESA study [4, 29]. There was a 36.7% incidence of CIMT >0.9 mm reported by Mitu *et al*. [28] among apparently healthy individuals, classified mainly in high risk SCORE, and an incidence of 34% was reported by Novo *et al*. [37] in an older study group, with a relatively high prevalence of diabetic and hypertensive patients.

Similarly to our results, the 75th percentile of the CCA-IMT distribution was established at 0.58 and 0.59 mm in healthy females and males without CV RFs, over 40 years of age [38, 39]. In a recent study of an apparently healthy population aged 57.7 ± 10.4 years, without exclusion of DM, the distribution of pathological CIMT >0.74 mm (75% percentile) was 25.96% (lower than in our study), but it followed a higher cut-off level in comparison with our study [40]. The prevalence of CIMT >75th percentile for the patient’s age, sex and race/ethnicity was approximately 12% across the Framingham study, but at intermediate FRS, 22–58% of patients had increased CIMT [41]. However, no data are available on the progression rate of pathological age- and sex-adjusted CIMT in the literature.

### 5.3 Carotid Plaque Progression

USG measures of carotid IMT and plaque are non-invasive methods for measuring ATS burden and strongly associated with vascular RFs and the incidence of CV events [35]. ATS progression predicts CV events [14]. The occurrence of carotid plaques seems to be variable in the general population and might be explained by geographical influence, age and the presence of CV RFs [28]. According to a systematic review [7], the occurrence of plaque in asymptomatic, low-to-intermediate risk cohorts, with different age and risk profile was an average of 35% (from 1.4% to 65.3%). Some studies [11, 28, 37, 40] in comparison to our results, reported a higher prevalence of carotid plaque (78%, 40%, 25%, 34%, resp.) probably due to the enrollment of older subjects. Data from studies with asymptomatic, middle-aged individuals documented higher occurrence of carotid plaques (29.3% in subjects with risk SCORE <5% [28], 31% in the PESA Study [29]). In the Refine study among 50–69-year-old participants, after a 4.2-year follow-up, in those patients without plaque at the first visit, the rate of plaque burden was 29.7%, which is a higher progression than in our study, but in a population with worse risk profile, with no exclusion of CVD [32]. Similar to our data, 20.5% of subjects developed new carotid artery plaques during a 5-year follow-up in a community in Taiwan (older subjects, no exclusion of DM) [16].

### 5.4 Association of CIMT with Cardiovascular Risk Factors

CIMT is associated with CVD RFs, the prevalence and incidence of CVD, and the degree of ATS in several different arterial beds [42]. In line with various studies in healthy populations, we documented associations between almost all classical CV RFs and CIMT parameters, however, in univariate analysis the associations were dominantly weak (datasets from univariate analysis are available from the corresponding author on request). Some studies showed robust correlation between age and the CIMT [43, 44]. In the Happy study, it was relatively better for the female cohort, which is partially in line with our findings [43]. Although the CIMT is thicker in men [26, 36, 38], sex does not independently predict the CIMT [45]. In other small cross-sectional studies of healthy subjects, age, BMI, waist circumference, systolic blood pressure (SBP) and diastolic blood pressure as well as TAG, HDL-C, glycaemia, and histories of CVD and type 2 DM [40, 44] were significantly associated with CIMT. Central obesity was significantly associated with CIMTmax and mean CIMT, while AH was only associated with CIMTmax in our study. There are non-consistent results in the literature regarding CIMT and lipoproteins. Most single-centre studies indicate the relationship between higher CIMT and higher levels of T-C, LDL-C, and non-HDL-C, as well as inverse associations with HDL-C [44, 45] (comparable to us); however, meta-analyses fail to show any associations [46, 47]. Similar controversies in association between HbA1c and the CIMT were revealed in non-diabetic individuals [48]. Alizargar *et al*. [44] showed a significant and strong correlation between HbA1c and CIMT, which we confirmed for MetS. With an increasing number of RFs, the increase of mean IMT in all carotid arterial segments was found in the Bogalusa Study [49], we confirmed weak associations between risk SCORE and mean as well as maximum CCA IMT values.

In the multivariate analysis, age appeared to be the most common independent predictor of CIMT [44, 45]. Apart from age, Alizargar *et al*. [40] also found, that waist circumference, SBP and C-reactive protein (CRP), and Paul *et al*. [49], also found that male sex, T-C/HDL-C ratio and smoking were common independent predictors of CIMT. Mitu *et al*. [28, 50] reported, that risk SCORE positively, significantly and also independently correlated with CIMT and the presence of carotid plaques in a small, clinically healthy, middle-aged cohort. In contrast, we found only a weak prediction of mean CIMT with T-C, and CIMT max with risk SCORE, but we found no positive influence of other RFs on CIMT. This is probably due to the limited range of age and our small study sample.

In a univariate analysis by Novo *et al*. [37], CIMT ≥0.9 mm or carotid plaque presence were related to the major CV RFs (age, AH, DM, HDL-C) and were independently associated with a major incidence of cerebro- and CV events. Similar data were published from a meta-analysis of 75 studies on the increased CIMT >1 mm and carotid plaque incidence with CV RFs [46]. In other studies age and waist circumference were predictors of high CIMT after adjustment for confounders [40, 45], with waist circumference being the strongest independent predictor [44]. Individuals with CIMT values >0.9 mm have a 4.1 times higher risk for being at a high SCORE CVD risk in a multivariate analysis [28]. We did not confirm any impact on CIMT >0.9 mm probably due to its low prevalence. In our subjects with central obesity there was 2.4× higher probability of CIMT detection over the age-and sex-specific cut off level, but after adjustment for confounders, the association disappeared. There are no comparable data in the literature.

### 5.5 Association of Carotid Plaque with Cardiovascular Risk Factors

Carotid ATS (IMT, plaques) is independently associated with all traditional RFs and CVD [35, 51]. Thickening of the CIMT reflects early stages of ATS, but plaque formation indicates later stages [52]. A meta-analysis of 76 cross-sectional studies with evaluation of 11 RFs, showed an association between the incidence of carotid plaque and AH, DM, DLP, current smoking, hypertriglyceridemia, LDL-C, hyperuricemia, hyperhomocysteinemia, and MetS [47]. We did not analyse the impact of DM and hyperhomocysteinemia, but found similar significant associations of RFs (also central obesity and SCORE risk) with the occurrence of carotid plaque. In line with our findings, Mitu *et al*. [50] documented an association of increased CV risk scores with the presence of carotid plaque and suggested the screening of subclinical ATS in subjects with a risk SCORE ≥3. Sex, T-C, LDL-C, TAG, non-HDL-C, waist, smoking and CVD risk score were associated with the risk of plaque formation in plaque-free subjects at baseline, however, in multivariable analyses, LDL-C was the only RF associated with plaque formation [32]. In addition, in the study from Taiwan, subjects with new carotid artery plaques during follow-up were older, hypertensive, and diabetic, but there was no association after controlling for CV RFs [16]. In asymptomatic individuals without DM, a positive association between HbA1c levels (also bellow the pre-diabetes cut off range) and subclinical ATS was identified even after adjustment for potential confounders (except for T-C) [53]. Fasting glucose levels showed a positive association with the prevalence and extent of subclinical ATS in univariate, but not in multivariate analysis [53]. In the PESA study, all traditional RFs as well as ATS CVD 10-year risk contributed to the progression of subclinical ATS across coronary and multiple noncoronary territories in the univariate analysis. In contrast, only age, male sex, and DLP were significantly associated with new ATS onset in disease-free participants [29]. Age, male sex, and all other CV RFs except obesity, DM, and estimated risk showed an independent association with ATS progression in multivariate analysis, with DLP as the strongest modifiable RF [29]. In our study, the only significant predictor of the presence of carotid plaque was the number of RFs. We only assessed the carotid region and analysed the correlation between the RFs and the occurrence of carotid plaque at the end of follow-up.

### 5.6 Personalized Cardiovascular Risk Assessment

The presence of subclinical ATS is the major causal RF for CVD in asymptomatic individuals rather than a prominent additional predictive factor [54]. Carotid plaque burden may detect early stages of disease even before coronary calcification [11]. The Multi-Ethnic Study of Atherosclerosis (MESA) study showed that adding different plaque metrics with CIMT measurements to RFs significantly increased the association with the incidence of CVD events [55]. The BioImage study showed a significantly higher risk prediction performance of manual three-dimensional (3D) quantification of plaque thickness compared with two-dimensional (2D) measurements of plaque and CIMT [3]. A first clinical event in the 10 years follow-up was reported in 32% of subjects with carotid wall thickening and 62% with asymptomatic carotid plaque, moreover carotid subclinical ATS was related to the major CV RFs enhancing the predictive value of risk scores especially in the low- risk population [37]. Similarly, in the Heinz Nixdorf Recall (HNR) study CAC, CIMT, and ankle-brachial index (ABI) were associated with stroke in addition to established RFs [56]. CV disease primary prevention guidelines prioritize risk stratification by using clinical risk scores; beyond traditional RFs, CAC scoring, or the presence of carotid plaque as a high risk finding [1]. USG are sufficient for determining an accurate prediction of the CV risk in asymptomatic patients. Carotid USG results should be combined with other ATS factors, and a comprehensive risk assessment may help to guide CV prevention decisions. The observation in the Refine study that risk scores are predictive of new plaque formation in patients with no plaque at the first visit [32], the proposal of Mitu *et al*. [50] to screen populations with a risk SCORE ≥3, the characteristics of a screened population with ≥3 RFs for measurement of subclinical vascular disease with the aim of improving CV risk prediction [57], in line with our conclusions, may be of value to determine who should be given more attention to modify or treat individual RFs.

## 6. Limitations

Limitations of our study are: a small number of participants, lower response rate (75%), low prevalence of some morphological markers, effect of collinearity, as well as the elimination of incomplete results, which weaken the statistical power in sub-analyses. Moreover, the lack of methodological standardization, measurement difficulties and publication bias make it difficult to compare our results with other studies. In addition, there are limited data focusing on the progression of subclinical ATS in similarly selected subjects and using markers. Due to these limitations, there is a need for cautious interpretation of our results. Additional research in a larger sample of asymptomatic individuals is needed to quantify the impact of imaging for subclinical ATS in CV risk management before applying them in clinical practice.

## 7. Conclusions

In middle-aged, non-diabetic, low-to moderate CV risk individuals, during a short follow-up, a relatively high prevalence and significant progression of subclinical carotid ATS was detected by widely available, non-invasive, standardized ultrasound techniques, expressed mainly as the presence of carotid plaque and age- and sex-adjusted increase of CIMT. The number of classical RFs independently predicted the occurrence of carotid plaque and was a determinant of CIMT progression. The high prevalence and short-term progression of subclinical carotid ATS (between 45 and 50 years of patients’ age), in addition to the evidence based predictive power of ATS burden on the incidence of CV events, may underline the rationale for carotid ATS screening and personalized CV risk stratification in middle-aged subjects with low-to moderate calculated CV risk, especially in those over 50 years old with several RFs.

## Data Availability

The datasets generated and analysed during the current study are not publicly available due to the institution policy but are available from the corresponding author on reasonable request.
